# Total Psoas Area and Total Muscular Parietal Area Affect Long-Term Survival of Patients Undergoing Pneumonectomy for Non-Small Cell Lung Cancer

**DOI:** 10.3390/cancers13081888

**Published:** 2021-04-14

**Authors:** Elisa Daffrè, Mathilde Prieto, Katharina Martini, Trieu-Nghi Hoang-Thi, Nara Halm, Hervè Dermine, Antonio Bobbio, Guillaume Chassagnon, Marie Pierre Revel, Marco Alifano

**Affiliations:** 1 Department of Thoracic Surgery, Paris Centre University Hospitals, AP-HP, 75014 Paris, France; elisa.daffre@aphp.fr (E.D.); mathilde.prieto@aphp.fr (M.P.); antonio.bobbio@aphp.fr (A.B.); 2 Department of Diagnostic and Interventional Radiology, University Hospital Zurich, Rämistrasse 100, CH-8091 Zurich, Switzerland; katharina.martini@usz.ch; 3 Department of Radiology, Paris Centre University Hospitals, AP-HP, 75014 Paris, France; thi-trieu-nghi.hoang@etu.u-paris.fr (T.-N.H.-T.); narahalm11@gmail.com (N.H.); guillaume.chassagnon@aphp.fr (G.C.); marie-pierre.revel@aphp.fr (M.P.R.); 4 Department of Anesthesiology and Intensive Care, Paris Centre University Hospitals, AP-HP, 75014 Paris, France; herve.dermine@aphp.fr; 5 Faculty of Medicine, University of Paris, 75006 Paris, France

**Keywords:** sarcopenia, pneumonectomy, lung neoplasms, survival

## Abstract

**Simple Summary:**

Lung cancer continues to be one of the leading cause of cancer-related deaths. In multimodality management of non-small cell lung cancer, surgery remains the mainstay, and in particular, pneumonectomy remains the only possible surgical procedure in patients with centrally located lesions. However, despite improvements in technique and perioperative management, it continues to be associated with significant postoperative mortality. Thus, identifying patients at high postoperative risk is of paramount importance to select surgical candidates, but identifying patients more likely to achieve definitive cure after surgery is at least as important. Among all the evaluated parameters, we found that being sarcopenic at both psoas and parietal muscles is an independent negative prognostic factor of overall survival. The whole muscular area had the best predictive value among all of the tested factors evaluating sarcopenia.

**Abstract:**

There is no standardization in methods to assess sarcopenia; in particular the prognostic significance of muscular fatty infiltration in lung cancer patients undergoing surgery has not been evaluated so far. We thus performed several computed tomography (CT)-based morphometric measurements of sarcopenia in 238 consecutive non-small cell lung-cancer patients undergoing pneumonectomy from 1 January 2007 to 31 December 2015. Sarcopenia was assessed by the following CT-based parameters: cross-sectional total psoas area (TPA), cross-sectional total muscle area (TMA), and total parietal muscle area (TPMA), defined as TMA without TPA. Measures were performed at the level of the third lumbar vertebra and were obtained for the entire muscle surface, as well as by excluding fatty infiltration based on CT attenuation. Findings were stratified for gender, and a threshold of the 33rd percentile was set to define sarcopenia. Furthermore, we assessed the possibility of being sarcopenic at both the TPA and TPMA level, or not, by taking into account of not fatty infiltration. Five-year survival was 39.1% for the whole population. Lower TPA, TMA, and TPA were associated with lower survival at univariate analysis; taking into account muscular fatty infiltration did not result in more powerful discrimination. Being sarcopenic at both psoas and parietal muscle level had the optimum discriminating power. At the multivariable analysis, being sarcopenic at both psoas and parietal muscles (considering the whole muscle areas, including muscular fat), male sex, increasing age, and tumor stage, as well as Charlson Comorbidity Index (CCI), were independently associated with worse long-term outcomes. We conclude that sarcopenia is a powerful negative prognostic factor in patients with lung cancer treated by pneumonectomy.

## 1. Introduction

In spite of advances in multimodality management of non-small cell lung cancer, surgery remains the mainstay [1]. Surgical techniques have been refined in the past decades to allow parenchymal-sparing procedures, including bronchial and bronchovascular sleeve resections [2], but pneumonectomy continues to be the sole possible surgical procedure in a subset of lung cancer patients with centrally located lesions [3]. Pneumonectomy continue to be associated with significant postoperative mortality [4,5,6], with figures around 10 percent; thus, identifying patients at high postoperative risk is of paramount importance to select surgical candidates [4,5,6], but identifying patients more likely to achieve definitive cure after surgery is at least as important [3]. As a general rule in lung cancer, pathologic stage is generally considered as the most powerful determinant of long-term survival [7]. However, patients undergoing pneumonectomy have often locally advanced disease, reducing the interest of tumor stage as the dominant outcome determinant [3,4,5,6,7,8]. In the era of patient-directed thinking, rather than cancer-directed thinking, assessing host parameters seems particularly promising [9].

In a series of 161 consecutive pneumonectomy patients, we have previously showed that sarcopenia (defined as sex-specific total psoas area [TPA] at the 3rd lumbar vertebra level below the 33rd percentile), was an independent negative prognostic factor of long-term survival [8]. Since that publication, there has been accumulating evidence that, in cancer patients (regardless from the kind of cancer and administered treatments), sarcopenia can be better assessed by taking into account the other abdominal muscles (Total Muscular Parietal Area, TMPA) at the same level (L3), thus calculating Total Muscular Area (TMA; TMA = TPA + TMPA); TMA would have better prognostic discrimination than TPA [10,11,12]. Furthermore, technological evolutions allow easy calculation by a computed tomography-scan (CT-scan) of muscular fat content, allowing to take into account only the muscular part of each muscle, in the idea that muscular fat infiltration (i.e., myosteatosis) could impact the outcome [11,12]. Effectively, we have recently shown that in patients undergoing pneumonectomy, fat-excluded TMA at the level of the third lumbar vertebra was the most discriminating parameter in predicting postoperative respiratory failure, Acute-Respiratory Distress Syndrome (ARDS) and in-hospital as well-as 30-day mortality [11]. However, fat is not necessarily a negative prognostic determinant of long-term survival in lung cancer patients (the so-known “lung cancer paradox”) and the prognostic impact of muscular fat infiltration needs to be assessed [8,9,10,11,12,13].

Thus, in the present study, we aimed (1) to assess in a larger series of consecutive patients undergoing pneumonectomy for lung cancer, which CT-based morphometric measurements of sarcopenia best predicts long-term overall survival; (2) to study correlations between different CT-based morphometric measurements of sarcopenia and main clinic-pathologic parameters. Thus, we collected both crude and corrected (by fat-attenuation) muscle areas, performed clinic-pathological correlations, and assessed the prognostic impact of cross-sectional psoas and parietal muscle areas, considered alones or together as a whole.

## 2. Material and Methods

### 2.1. Patients

**Figure 1:**
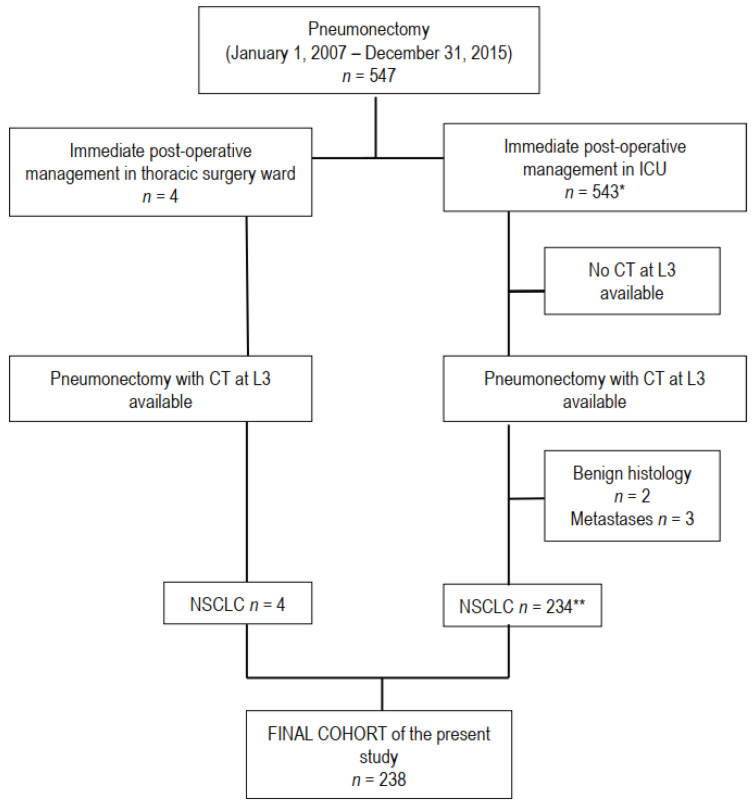
Flow chart of the study. Computed tomography (CT), L3 level of third lumbar vertebra, malignant pleural mesothelioma (MPM), number of patients. * n° 4 and 5 in reference list; ** n° 11 in reference list.

The hospital information system was retrospectively searched for consecutive patients who underwent pneumonectomy for non-small cell lung cancer by our surgical team between 1 January 2007 and 31 December 2015. Inclusion criteria were non-small cell lung cancer treated with pneumonectomy and availability of a preoperative CT-scan in the Picture Archiving and Communication System (PACS) of the hospital. The flow chart of the study is showed in Figure 1. The patient population overlapped almost completely that of a previous study evaluating the impact of sarcopenia on the occurrence of postoperative complications of pneumonectomy [11]. Of note, the Thoracic Surgery Department of the Paris Centre University Hospital is a high-volume tertiary referral center, performing each year approximately 1600 surgical procedures, half of them being major lung resections for non-small cell lung cancer (NSCLC). Patients are referred by several pulmonologist or oncologist teams from different hospitals in (and outside) the Paris area.

The research was conducted according to recommendations outlined in the Helsinki Declaration. The Institutional Review Board approval was obtained (CERC-SFCTCV-2015-11-4-13-16-16-AlMa).

All patients underwent routine preoperative work, together with thoracic and upper abdominal (see below) CT scan contrast-enhanced cerebral CT scan, or Magnetic Resonance Imaging (MRI), as well as fiber optic bronchoscopy. The 18-Fluordesoxyglucose CT coupled positron emission tomography was also routinely employed. Invasive mediastinal staging (endobronchial ultrasounds-transbronchial needle aspiration or mediastinoscopy/video-assisted thoracoscopy) was indicated in case of enlarged (short-axis > 1 cm) and/or hypermetabolic lymph nodes. Patients with proven N2 disease underwent platinum-based neoadjuvant chemotherapy followed by surgery in case of response or stable diseases. Adjuvant radiotherapy or chemotherapy was proposed on an individual basis under the care of the referring pulmonologist or oncologist, following multidisciplinary guidelines and evidence-based discussions. Moreover, postoperative adjuvant treatments and follow-up measures are not standardized among the different referring centers.

### 2.2. Collected Data

A standardized case report form was employed to collected patient characteristics, treatment procedures, and short-term outcomes, as previously described [6,7,8,9]. In particular, we collected: age, sex, weight, height, Body Mass Index (BMI), smoking habits, side of tumor, comorbid illness, Charlson Comorbidity Index (CCI), American Society of Anesthesiologists score (ASA score), respiratory function parameters (forced expiratory volume (FEV1), FEV1/forced vital capacity (FVC) ratio, carbon monoxide transfer coefficient (KCO), predicted postoperative FEV1, modified Borg dyspnea scale). We also collected induction treatments, histologic type, and pathologic stage. In particular, stage was re-attributed according to the 8th edition of the Tumor Node Metastasis (TNM) classification [14]. Postoperative outcome was recorded, in the particular occurrence of postoperative respiratory failure requiring mechanical ventilation, post-pneumonectomy ARDS, circulatory failure requiring inotropic drugs, and in-hospital or 30-day mortality. Late vital statuses of patients were checked by the Institute National Statistique et Economie, National Institute for Statistics and Economy (INSEE) website [15].

### 2.3. CT Scan

As previously described [11], all included patients had routine preoperative CT scans on 16- to 64-detector CT units from different vendors at tube voltages of 100 to 140 kVp, with or without contrast media injection. Images were reconstructed with a soft tissue convolution kernel at slice thicknesses from 0.75 to 3.0 mm.

### 2.4. Sarcopenia

For each patient, two radiologist fellows identified a slice in the axial plane at the L3 level, in which both vertebral transverse processes were fully visible. Subsequently, the following measurements where obtained: (1) total psoas area (TPA) comprising the cross-sectional area of the right and left psoas, (2) total parietal muscle area (TPMA), comprising the cross-sectional area of the skeletal muscles, excluding the psoas muscle. Total muscle area (TMA) was obtained from the sum of the former two.

In order to obtain values of the pure muscle area, excluding fatty degeneration, the same measurements were performed using an attenuation threshold of −29 to 150 Hounsfield units, allowing calculation of Fatty Excluded TPA and TPMA (FE-TPA and FE-TPMA, respectively), whose sum allowed estimation of Fatty Excluded TMA (FE-TMA) [11].

Height normalized values of TPA, TPMA, and TMA as well of FE-TPA, FE-TPMA, and FE-TMA were obtained for each patient and further referred to as the skeletal muscle index (SMI). SMI was calculated as follows: SMI = muscle area/height × height. As previously reported [9], sarcopenia can be defined as less than the sex-matched 33rd percentile of the assessed muscle area. Thus, we estimated sarcopenia at psoas (TPA and FE-TPA) and Parietal Muscle Area (TPMA and FE-TPMA), as well at total muscle area TMA (TMA and FE-TMA). Furthermore, as distribution TPA and FE-TPA did not exactly parallel that of TPMA and FE-TPMA (i.e., a patient could be sarcopenic with respect to psoas but not parietal muscles and vice versa), we assessed the possibility of being sarcopenic, or not, on both sites, by taking into account (FE-SARC) or not fatty exclusion (SARC).

### 2.5. Statistical Analysis

Data processing and analysis were performed with the statistical software system SEM (SILEX Development, Mirefleurs, France). Results were expressed as percentage, mean ± SD for normally distributed and median (interquartile range) for non-normal distributed quantitative variables.

Univariate analyses were performed to assess relationships with patients’ characteristics and histological features, and correlations were assessed by the Spearman rank test for continuous variables.

Survival analyses were performed by Kaplan–Meier and comparisons by Log-rank. Factors identified as associated with overall survival at univariate analysis were entered into the Cox multivariable models to assess their independent prognostic value. A step-by-step backward approach was used. Statistical significance was accepted at *p* < 0.05.

Furthermore, logistic regression was performed to search factors independently associated to being alive 5 years after the operation; a model that included only factors identified at univariate analysis was built.

## 3. Results

### 3.1. Patient Population

A total of 238 patients met all inclusion criteria and were identified for the retrospective analysis of our study (Table 1). Pneumonectomy with radical node dissection was carried out through a standard thoracotomy in all cases (right, *n* = 108; left, *n* = 130; 45.4% and 54.6%, respectively). Neoadjuvant chemotherapy (*n* = 71; 30.2%) was administered in patients with pathologically confirmed N2 disease. Meanwhile, between CT and surgery, it was 16 ± 20 days.

### 3.2. Morphometric Measurements

We showed that inter-reader-agreement was excellent for TPA, TPMA, and TMA with an interclass correlation coefficient (ICC) ranging from 0.81 to 0.96 [11]. Mean TPA, TPMA, and TMA at the L3 level were 16.6 ± 5.3 cm^2^, 131.8 ± 29.9 cm^2^, and 148.4 ± 34.0, respectively; details with respect to sex are shown in Table 2. Fat-exclusion allowed determination of FE-TPA, FE-TPMA, and FE-TMA; these figures being on the average 15.9 ± 5.1 cm^2^, 118 + 0 ± 26.5 cm^2^, and 133.9 ± 30.5 cm^2^, respectively (Table 2). Men had significantly higher areas than women and the difference persisted after fat-exclusion (*p* < 0.0000001), as well as after normalization by square height (SMI) (*p* < 0.0000001) (Table 2). Thus, we set cut-offs for further analyses, for each muscle or muscular group, as a whole or after fat exclusion, at the 33rd percentile in each sex, in agreement with previous studies [8,9,10,11]: different cut-offs are thus showed in Table 2. Furthermore, as we observed that sarcopenia, at either psoas or parietal muscles (with fat exclusion or not), was not always associated to sarcopenia in parietal muscles (and vice versa), we determined the number of patients with sarcopenia involving all of the different muscular groups or not (Table 2). Thus, 36 out of 169 men and 11 out of 69 women had sarcopenia (without fat exclusion), involving both psoas and parietal muscles; these figures were 36/169 men and 13/69 women when considering fat exclusion (Table 2).

Table 3 shows the distribution of sarcopenia (according to different muscles or muscular groups), with respect to main clinical and pathologic characteristics. Sarcopenia (estimated without fat exclusion) was more frequent in older patients, those having lower body weight, lower BMI, or higher Charlson Comorbidity Index (CCI). When sarcopenia was estimated taking into account fat exclusion (Table 4), it was more frequent in older patients and in those with lower body weight or BMI.

### 3.3. Outcome

Postoperative mortality rate was 9.0%. Postoperative respiratory failure requiring mechanical ventilation, post-pneumonectomy ARDS, and postoperative circulatory failure requiring inotropic support occurred in 15.7%, 9.4%, and 13.8% of patients. Factors (including sarcopenia) associated to occurrence of postoperative complications after pneumonectomy have been previously reported [11]. All of the patients surviving the operative period were available for late follow-up. INSEE register was interrogated on 14 February 2021, i.e., >5 years after the last operated patient.

**Figure 2:**
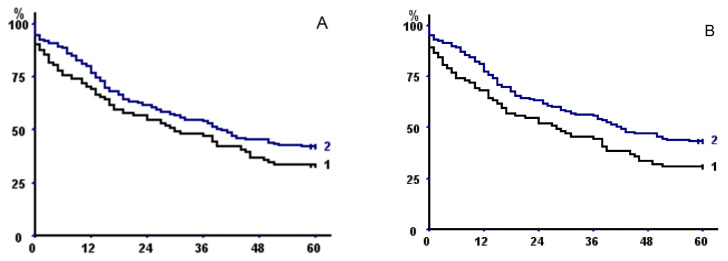

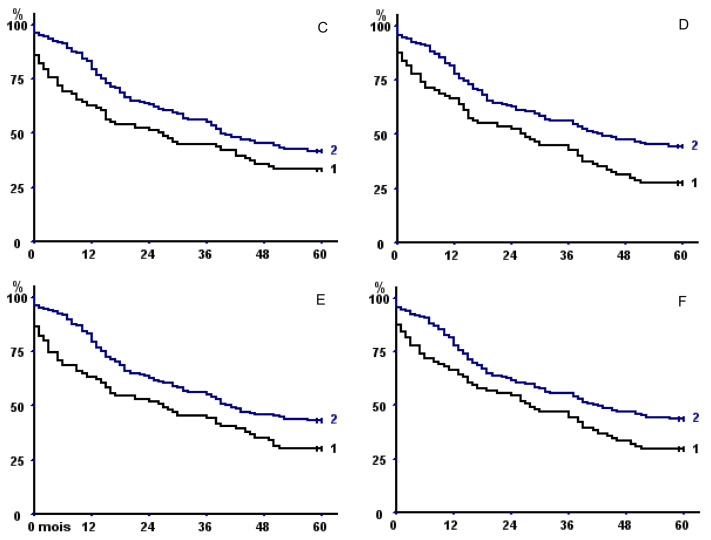
Kaplan–Meier survival curves in the whole population with respect to (**A**) psoas area with fat exclusion, (**B**) total psoas area, (**C**) parietal area with fat exclusion, (**D**) total parietal area, (**E**) total muscle area with fat exclusion, and (**F**) total muscle area. In all of the panels, curve 2 represents patients with sex-specific areas greater than the 33rd percentile versus less than or equal to the 33rd percentile (curve 1).

Univariate analysis of factors affecting long-term survival is shown in Table 5. Log-rank showed that increasing age, male sex, underweight, higher CCI, and higher pathologic stage were unfavorable prognostic factors. Sarcopenia (defined as TPA, TPMA, or TMA) had a negative prognostic impact (Table 6, Figure 2); generally, measures of the whole muscle performed better that FE measures.

**Figure 3:**
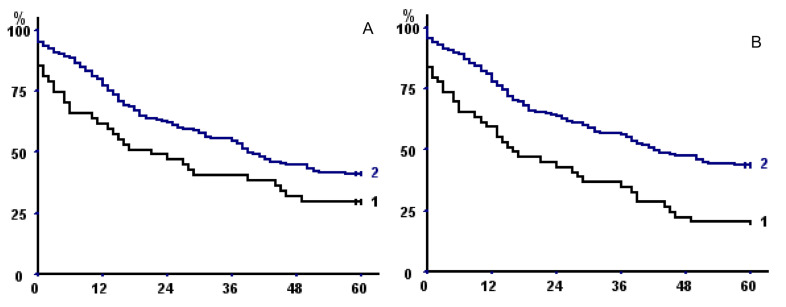

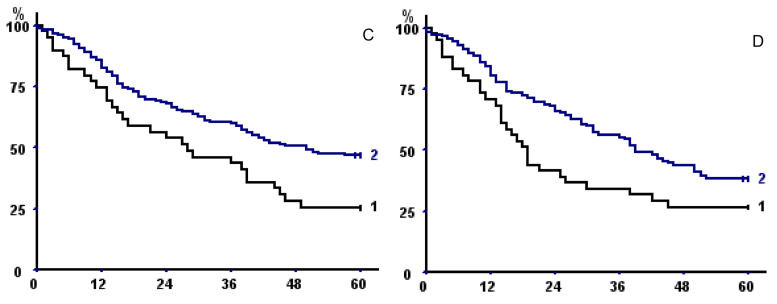
(**A**,**B**) Kaplan–Meier survival curves in the whole population with respect to (**A**) fat excluded sarcopenia of both psoas and parietal muscles and (**B**) sarcopenia of both psoas and parietal muscles (without fat exclusion). In both panels, curve 1 represents sarcopenic patients; curve 2 non-sarcopenic. (**C**) Kaplan–Meier survival curves with respect to sarcopenia of both psoas and parietal muscles (without fat exclusion) taking into account only operative survivors. (**D**) Kaplan–Meier survival curves of male operative survivors with total muscular fat <25th percentile (1) versus >25 percentile (2).

TMA had a better discriminating power than TPA or TPMA, but discrimination was even better when taking into account sarcopenia at both psoas and parietal muscle areas (Table 6), both without fat exclusion (*p* = 0.00024) (Figure 3A) or with (*p* = 0.041) (Figure 3B).

Two stepwise Cox models were built to assess the independent prognostic value on overall survival of factors identified at univariate analysis, one introducing total sarcopenia (on both psoas and parietal muscles), the other TMA (Table 7): we entered in both models sex, age (≤64 vs. >64 years, which represents the median value), CCI (≤5 vs. >5, which represents the median value), BMI (underweight vs. others), and pathologic stage (four classes: I–II, IIIA, IIIB, and IV); total sarcopenia (on both psoas and parietal muscles), and TMA were entered in the first and the second models, respectively. The first model allowed to affirm the independent prognostic value of the pathologic stage (Relative Risk (RR) I–II: reference (REF), IIIA: 1.39 (1.14–1.70), IIIB: 1.93 (1.29–2.89); IV: 2.69 (1.47–4.92), *p* = 0.0013), male sex (RR 1.95 (1.30–2.92), *p* = 0.0012), sarcopenia (RR 1.71 (1.17–2.52), *p* = 0.006), and higher age (RR 1.48 (1.06–2.06), *p* = 0.04). The second model showed that male sex (RR 1.89 (1.26–2.85), *p* = 0.0022), higher pathologic stage (RR: I–II: REF, IIIA: 1.39 (1.14–1.70), IIIB: 1.94 (1.30–2.89), IV: 2.70 (1.49–4.91), *p* = 0.0011), TMA below the sex-specific 33th percentile (RR: 1.54 (1.10–2.16), *p* = 0.0012), and Charlson Comorbidity Index >5 (RR: 1.51 (1.08–2.10) *p* = 0.0015) independently predicted outcome. In both models, the independent prognostic values of the same factors were also evident before step-by-step elimination of non-significant factors.

Of note, when taking into account only operative survivors, we confirmed the negative prognostic impact of sarcopenia of both psoas and parietal muscles on long-term outcome (*p* = 0.04, Figure 3C).

We also assessed the impact of different SMI measures (TPA, TPMA, or TPA, fatty excluded or not) on survival. The discriminating power was inferior for all the tested variables (not shown); for example, when using SMI fatty-excluded TMA at cut-off (showed in Table 2 for men and women), we observed that 5-year overall survival was 31.6% (22.45–42.55) in sarcopenic and 42.6% (35.07–50.45) in non-sarcopenic patients, respectively (*p* = 0.047).

### 3.4. Long-Term Survivors

A total of 93 patients (39 women, 54 men) were alive more than 5 years after the operation. Being alive more than 5 years after pneumonectomy was associated at univariate analysis to the female sex (*p* = 0.00042), lower age (*p* = 0.046), lower CCI (*p* = 0.0017), absence of sarcopenia at both psoas and parietal muscles (measures without fat exclusion) (*p* = 0.0027). Logistic regression (model built by introducing the above identified factors) showed that female sex (Odd Ratio (OR) 2.37 (1.26–4.44), *p* = 0.0073), CCI ≤ 5 (OR 2.06 (1.12–3.81), *p* = 0.021), and absence of sarcopenia at both psoas and parietal muscles (OR 2.70 (1.19–6.13), *p* = 0.018) were independent protective factors.

### 3.5. Fat: Exploratory Analysis

To understand the pathophysiological role of fatty muscular infiltration, we assessed clinical–pathological correlations of total muscular fat (TMA–FETMA) and its prognostic impact.

Mean total muscular fat area at L3 level was 13.94 ± 8.31 cm^2^ (14.93 + 8.32 in men, 11.51 + 7.75 in women, *p* = 0.0018). Total muscular fat area was directly correlated with TMA (r = 0.274, *p* = 0.000037), BMI (r = 0.564, *p* < 0.0000001), and KCO (r = 0.297, *p* = 0.0028), but also with age (r = 0.289, *p* = 0.000015) and CCI (*p* = 0.00047); it was lower in patients who had stopped tobacco consumption (14.64 ± 8.18 vs. 11.4 ± 6.77, *p* = 0.0075). Exploratory analysis showed that total muscular fat below the 25th percentile was associated to shorter long-term survival in men who survived the operative period (*p* = 0.033) (Figure 3D).

### 3.6. The Impact of Postoperative Complications on Long-Term Outcome

**Figure 4:**
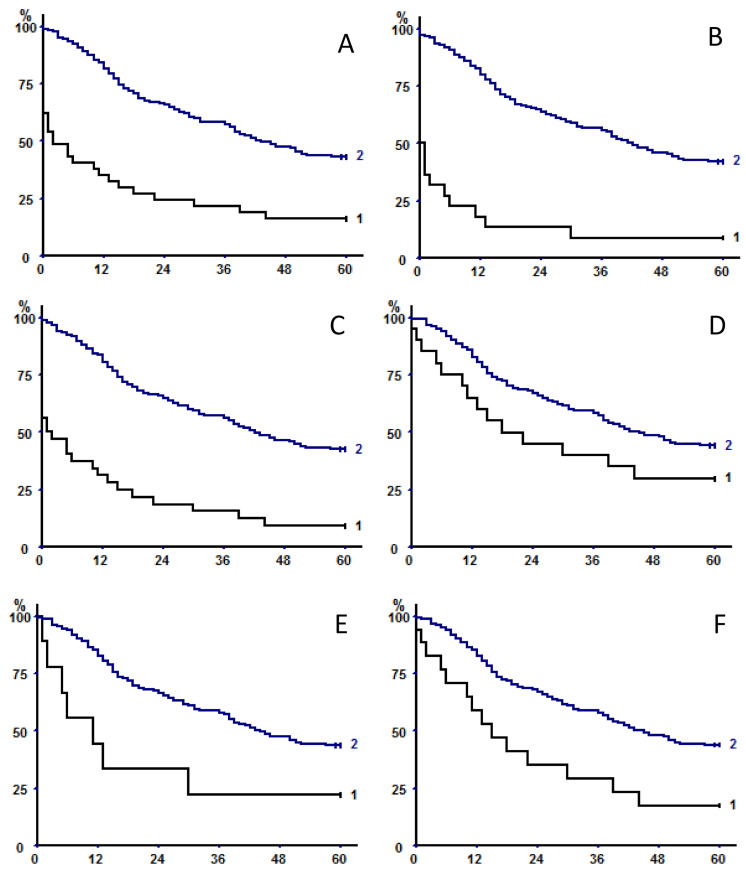
Long-term survival with respect to the occurrence (curve 1 in each panel) or not (curves 2) of major postoperative complications: respiratory failure requiring mechanical ventilation (5-year OS 16.2% (7.65–31.14) versus 43.4 (36.72–50.4), (**A**), post-pneumonectomy ARDS (5-year OS 9.1% (2.53–27.82) versus 42.3% (35.81–48.97), (**B**), circulatory failure requiring inotropic support (5-year OS 9.4 (3.24–24.22) versus 42.7% (36.04–49.66), (**C**). All of these complications were associated with shorter overall survival (always *p* < 0.0000001). Differences in survival did not persist when restricting the analysis to operative survivors in patients who experienced respiratory failure (5-year OS 30% (14.55–51.9) versus 44.3% (37.52–51.35), *p* = 0.063, (**D**), but did persist in operative survivors experiencing post-pneumonectomy ARDS (5-year OS 22.2% (6.32–54.74) versus 43.9% (37.28–50.75), *p* = 0.011, (**E**), or circulatory failure (5-year OS 17.6% (6.19–41.03) versus 44% (37.22–51.09), *p* = 0.0019, (**F**).

Figure 4 shows long-term survival with respect to the occurrence of major postoperative complications (respiratory failure requiring mechanical ventilation (i.e., Figure 4A), post-pneumonectomy ARDS (i.e., Figure 4B), circulatory failure requiring inotropic support (i.e., Figure 4C)). All of these complications were associated with shorter overall survival (always *p* < 0.0000001). Differences in survival persisted also when restricting the analysis to operative survivors with respect to post-pneumonectomy ARDS (*p* = 0.011) (i.e., Figure 4E) or circulatory failure (*p* = 0.0019) (i.e., Figure 4F), but not with respiratory failure (*p* = 0.063) (i.e., Figure 4D).

## 4. Discussion

In the present study, we report evidence that sarcopenia is independently associated with unfavorable outcome in patients with non-small cell lung cancer undergoing pneumonectomy. Among all of the evaluated parameters, being sarcopenic at both psoas and parietal muscles was associated with the worst outcome; this was also observed among operative survivors, as well as after correcting for classical prognostic factors, including sex, age, tumor stage, and CCI. Interestingly, total muscle area (including fat) rather than muscle area without fat allowed the best discrimination; we could also show that muscular fat infiltration positively impacted long-term outcome of men who survived the operative period.

Although sarcopenia is often defined as loss of mass, quality, and function of the skeletal muscles [9,10,12], most studies do not take into account all of these parameters of sarcopenia. We showed that whole muscle bulk contributed to a favorable outcome; i.e., fat may be protective as well. This is not completely surprising in lung cancer, where higher BMI has been associated with survival in lung cancer patients undergoing all kinds of resections, the so-called “lung cancer paradox” [8,9,10,11,12,13]. Although we showed in a similar dataset that fat-excluded TMA, at the level of the third lumbar vertebra, was the most discriminating parameter to assess short-term-outcome in patients undergoing pneumonectomy (especially the risk of respiratory complications) [11], this should not be regarded as contradictory to present results: possibly respiratory failure (and need of mechanical intubation) is more dependent on muscular bulk and function than on muscular fat reserve, which is probably more protective on the long-term period.

It is impossible to say if sarcopenia is likely to be a direct determinant of prognosis, but it is an extremely powerful marker of patient frailty [16]. Walking speed or walking activity, and expression of patient frailty could be assessed by several other measurements, but CT-based morphomics offer the advantage of objective assessment, which is independent of conditions of measure [9]. Furthermore, all patients with lung cancer have a CT scan descending down to L3 levels, thus avoiding repeat examination. Our findings concerning the deeply negative impact of being sarcopenic at both psoas and parietal muscle levels (these muscles having different physiologic actions and, as a consequence, being differently solicited according to different lifestyles) underlines that morphomics can somewhat assess not only muscle bulk, but also extent and type of function.

### 4.1. Mechanisms

Systemic inflammation and impairment of nutritional status are frequent features of lung cancer [17,18,19]. These two factors are strongly correlated in patients undergoing surgery for lung cancer and both are related to scarce infiltration of tumor by immune cells [19]. Inflammatory status (pre-existent or concomitant with lung cancer), with subsequent increased energy consumption, contributes to malnutrition. In turn, catabolic processes secondary to inflammation, and reduced caloric intake, may be responsible for fat and muscle loss [13,14,15,16,17,18,19]. Other mechanisms have also been evoked, including an imbalance in anabolic and proteolytic pathways [20]. In cancer patients, sarcopenia is associated (if not in part responsible for) physical decline, loss of quality of life, increased toxicity from chemotherapy, and shorter survival. The combination of sarcopenia and cachexia are believed to be the direct cause of death in at least 20% of oncologic patients with advanced disease [21].

Sarcopenia in a surgical setting has been less extensively evaluated than in general oncology. Measurements of muscle area on cross-sectional images are a well-accepted surrogate for muscle loss and have been shown to correlate with morbidity and mortality after major abdominal, vascular, or cardiopulmonary surgery [9]. We have previously shown that low value of TPA was associated with poor long-term survival in patients treated by pneumonectomy for lung cancer [8], and that sarcopenia assessed by morphometric measurements (tricipital skin-fold thickness and brachial circumference) also predicted poor long-term survival of patients suffering from non-small cell lung cancer undergoing all kinds of resections [13]. Herein we provide evidence on better discrimination ability of more fine assessment of muscle mass, composition, and, indirectly, function.

Although, according to a recent European consensus, computed tomography and magnetic resonance imaging were considered the imaging modalities of choice for estimating muscle mass, definition criteria of sarcopenia is not entirely standardized [10]. As previously suggested [8,9,10,11], the threshold to define sarcopenia was set at the sex-related 33rd percentile of our population: this distribution-based approach is fair, but our values need to be generalized in multicenter studies. Some authors advocate the use of height normalized SMI at L3 [22]; in our experience, this approach resulted in less discriminating results.

### 4.2. Limitations

The present study has some limitations: (1) it is retrospective in nature. However a large number of patients were analyzed and their surgical and peri-operative management was standardized with no significant changes over time. (2) The study setting: this was a mono-institutional study performed in a tertiary referral surgical department, whose patients were referred by several pulmonologists or oncologist teams from different hospitals with non-uniform policies in postoperative management. (3) The main outcome of study was overall survival, not progression-free survival or cancer-specific survival. As our patients were referred by different hospitals, postoperative management (including follow-up measures) was not standardized, and this would result in inconsistent results when studying the time of possible relapse. However, for a severe disease, such as lung cancer requiring pneumonectomy, the study of overall survival is probably at least as important as other outcome measures: we adopted an objective outcome measure, easy to collect with few missing data. (4) In spite of a relatively high number of pneumonectomies, a subgroup analysis may not have sufficient power; thus, we performed survival analysis according to occurrence of postoperative complications, but further stratification according to the presence of sarcopenia, or not, in patients developing postoperative complications (to study the impact on survival of sarcopenia in patients presenting respiratory complications) was impossible because of the low number of effectiveness in each subgroups. (5) CT images were acquired with a tube voltage between 100 and 140 kVp. We acknowledge that fat-exclusion based on CT attenuation, was not corrected for the different acquisition parameters, such as tube voltage; however, the differences were not large enough to influence the results. (6) We assessed sarcopenia by CT measurements; ideally, the real measure of muscular function (by hand grip or 6-minunte walking test) would have added strength to our work. Obviously, the retrospective nature of our work precluded availability of these parameters.

In conclusion, our study strongly supports the concept that sarcopenia is an independent risk factor for the occurrence of postoperative complications and mortality after pneumonectomy. The main interest in recognizing sarcopenia as a risk-factor resides in its potential reversibility thanks to rehabilitation programs.

## Figures and Tables

**Table 1:** Main demographical, morphometric, clinical, and laboratory features of the population. Information for each feature was available for the whole sample (*n* = 238) if not otherwise specified.

Features	Total Sample = 238
Age	63 ± 10.3
Sex: Men/Women	169 (71%)/69 (29%)
Current/Never Smokers (*n* = 237)	217 (91.6%)/20 (8.4%)
Cumulative Tobacco Consumption (Pack/Years) (*n* = 220)	40.0 (30.0–53.8)
Right/Left side	108 (45.4%)/130 (54.6%)
Weight (kg)	70.4 ± 13.8
Height (cm)	169 ± 9.2
BMI (kg/m^2^)	24.7 ± 4.2; median 25
Obesity	30 (12.8%)
Underweight/Normal Weight/Overweight	204 (87.2%)
Diabetes, Yes/No (*n* = 235)	20 (8.5%)/215 (91.5%)
Hypertension, Yes/No (*n* = 235)	83 (35.3%)/152 (64.7%)
Ischemic Heart Disease, Yes/No (*n* = 235)	41 (17.5%)/194 (82.5%)
Charlson Comorbidity Index (*n* = 216)	5.2 ± 1.8; median 5.0
Baseline Modified Borg Dyspnea Scale >2 (*n* = 237) Yes/No	43 (18.1%)/194 (81.9%)
FEV1 (% of predicted) (*n* = 237)	78.7 ± 17.4
FEV1/FVC ratio (*n* = 234)	70.9 ± 13.1
Predictive Postoperative FEV1 (ppoFEV1) (*n* = 182)	50.37 ± 11.37
KCO (% of predicted) (*n* = 100)	79.9 ± 22.3
Induction Chemotherapy Yes/No (*n* = 235)	71 (30.2%)/164 (69.8%)
Induction Radiotherapy Yes/No (*n* = 235)	4 (1.7%)/231 (98.3%)
Histology (*n* = 238):	
Adenocarcinoma	76 (31.9%)
No-Adenocarcinoma	162 (68.1%)
Pathologic stage (*n* = 238)	
0	1 (0.4%)
I	19 (8%)
II	54 (22.7%)
III	155 (65.1%)
IV	9 (3.4%)
pT (*n* = 237)	
T0	2 (0.8%)
T1	23 (9.7%)
T2	63 (26.6%)
T3	97 (40.9%)
T4	52 (21.9%)
pN (*n* = 238)	
N0	54 (22.7%)
N1	95 (39.9%)
N2	89 (37.4%)
pM (*n* = 238)	
Mx	1 (0.4%)
M0	228 (95.8%)
M1	9 (3.8%)

BMI: Body Mass Index, *n*: number of cases, FEV1: Forced Expiratory Volume in one second, FVC: Forced vital Capacity, KCO: Carbon Monoxide Transfer Coefficient, pTNM: pathologic Tumor Node Metastasis stage.

**Table 2:** Morphometric measurements in the whole population, with respect to sex.

	Overall (Mean ± SD)	Men (Mean ± SD)	Women (Mean ± SD)	*p*-Value
*Muscle areas* (cm^2^)				
Total psoas area at L3	16.6 ± 5.3	18.5 ± 4.8	11.9 ± 2.7	<0.0000001
33rd percentile	14	16.2	10.6	
Total parietal muscle area at L3	131.8 ± 29.9	143.6 ± 25.6	103.0 ± 17.6	<0.0000001
33rd percentile	114.2	134.5	98.2	
Total muscle area at L3	148.4 ± 34.0	162.1 ± 28.8	114.9 ± 19.1	<0.0000001
33rd percentile	127.7	151.6	109.2	
*Muscle area with fat exclusion* (cm^2^)				
Psoas area at L3 with fat exclusion	15.9 ± 5.1	17.7 ± 4.8	11.5 ± 2.7	<0.0000001
33rd percentile	13.8	15.6	10.4	
Parietal area at L3 with fat exclusion	118.0 ± 26.5	128.8 ± 22.5	91.5 ± 13.9	<0.0000001
33rd percentile	101.6	120.3	87	
Total muscle area at L3 with fat exclusion	133.9 ± 30.5	146.5 ± 25.8	103.0 ± 15.6	<0.0000001
33rd percentile	115.6	136.7	97.6	
*Skeletal muscle index, SMI* (cm^2^/m^2^)				
SMI—psoas area at L3 with fat exclusion	5.5 ± 1.6	6.0 ± 1.6	4.5 ± 1.1	<0.0000001
SMI—total psoas area at L3	5.8 ± 1.6	6.2 ± 1.6	4.6 ± 1.1	<0.0000001
SMI—parietal area at L3 with fat exclusion	41.2 ± 8.1	43.5 ± 8.0	35.4 ± 4.7	<0.0000001
SMI—total parietal muscle area at L3	46.0 ± 9.2	48.5 ± 9.0	39.9 ± 6	<0.0000001
SMI—total muscle area at L3 with fat exclusion	46.7 ± 9.2	49.4 ± 9.0	39.9 ± 5.4	<0.0000001
33rd percentile	41.8	45.5	37.5	
SMI—total muscle area at L3	51.8 ± 10.3	54.7 ± 10.1	44.5 ± 6.8	<0.0000001
33rd percentile	46.7	50	41	
Sarcopenia on psoas and parietal muscle	47	36	11	0.35
Sarcopenia either psoas or parietal + neither psoas and parietal	191	133	58	
Sarcopenia on psoas and parietal muscle with fat exclusion	49	36	13	0.67
Sarcopenia either psoas or parietal with fat exclusion + neither psoas and parietal with fat exclusion	189	133	56	

L3: level of the third lumbar vertebra, SMI: skeletal muscle index, SD: standard deviation.

**Table 3:** Correlations of sarcopenia (according to the different muscles or muscular groups without fat exclusion) with different clinical and pathological parameters).

Features	Psoas Sarcopenia		*p*-Value	Parietal Muscle Sarcopenia		*p*-Value	Total Muscle Area Sarcopenia		*p*-Value	Sarcopenia of Both Psoas and Parietal Muscles		*p*-Value
	Yes	No		Yes	No		Yes	No		Yes	No	
Age	65.8 ± 9.2	61.5 ± 10.5	0.0015	66.9 ± 10.7	61.1 ± 9.6	0.000046	66.8 ± 10.9	61.1 ± 9.4	0.0000048	67.7 ± 9.0	61.8 ± 10.3	0.00023
Men	58 (71.6%)	111 (70.7%)	0.88	55 (70.5%)	114 (71.3%)	0.91	55 (69.6%)	114 (71.7%)	0.74	36 (76.6%)	133 (69.6%)	0.35
Women	23 (28.4%)	46 (29.3%)		23 (29.5%)	46 (28.8%)		24 (30.4%)	45 (28.3%)		11 (23.4%)	58 (30.4%)	
Smokers (*n* = 291)												
Yes	23 (31.1%)	33 (22.8%)	0.18	18 (25.8%)	38 (25.5%)	0.97	20 (28.2%)	36 (24.3%)	0.54	13 (31%)	43 (24.3%)	0.37
No	51 (68.9%)	112 (77.2%)		52 (74.3%)	111 (74.5%)		51 (71.8%)	112 (75.7%)		29 (69.1%)	134 (75.7%)	
Cumulative tobacco consumption	43.6 ± 22.1	43.6 ± 22.2	0.82	42.5 ± 23	44.1 ± 21.7	0.54	42.7 ± 23.0	44.1 ± 21.7	0.55	47.0 ± 23.8	42.9 ± 21.7	0.32
(Pack/Years) (*n* = 291)												
Right side	35 (43.2%)	73 (46.5%)	0.63	43 (55.1%)	65 (40.6%)	0.035	44 (55.7%)	64 (40.3%)	0.024	24 (51.1%)	84 (44%)	0.38
Left side	46 (56.8%)	84 (53.5%)		35 (44.9%)	95 (59.4%)		35 (44.3%)	95 (59.7%)		23 (48.9%)	107 (56%)	
Weight (kg)	63.6 ± 10.4	73.9 ± 14	<0.0000001	61.6 ± 11	74.7 ± 12.9	<0.0000001	60.8 ± 9.8	75.2 ± 12.9	<0.0000001	60.2 ± 10	72.9 ± 13.4	<0.0000001
Height (cm)	169.1 ± 8.5	168.9 ± 9.5	0.75	166.8 ± 9.7	170.1 ± 8.7	0.036	166.5 ± 9.5	170.2 ± 8.8	0.013	169.1 ± 9.0	168.9 ± 9.2	0.74
BMI (kg/m^2^)	22.5 ± 3.6	25.8 ± 4.1	<0.0000001	22.3 ± 3.8	25.8 ± 3.9	<0.0000001	22.1 ± 3.5	25.9 ± 3.9	<0.0000001	21.2 ± 3.4	25.4 ± 4.0	<0.0000001
Obesity												
Yes	2 (2.5%)	28 (18.2%)	0.00067	2 (2.6%)	28 (17.8%)	0.0011	1 (1.28%)	29 (18.6%)	0.00019	0	30 (16.0%)	0.0037
No	78 (97.5%)	126 (81.8%)		75 (97.4%)	129 (82.2%)		77 (98.7%)	127 (81.4%)		46 (100%)	158 (84.0%)	
Diabetes (*n* = 235)												
Yes	8 (10%)	12 (7.7%)	0.56	7 (9.3%)	13 (8.1%)	0.76	7 (9.2%)	13 (8.2%)	0.79	5 (10.9%)	15 (7.9%)	0.73
No	72 (90%)	143 (92.3%)		68 (90.7%)	147 (91.9%)		69 (90.8%)	146 (91.8%)		41 (89.1%)	174 (92.1%)	
Hypertension (*n* = 235)												
Yes	31 (38.6%)	52 (33.6%)	0.43	32 (42.7%)	51 (31.9%)	0.11	33 (43.4%)	50 (31.5%)	0.072	17 (37%)	66 (34.9%)	0.8
No	49 (61.6%)	103 (66.5%)		43 (57.3%)	109 (68.1%)		43 (56.6%)	109 (68.5%)		29 (63%)	123 (65.1%)	
Ischemic heart disease (*n* = 235)												
Yes	17 (21.2%)	24 (15.5%)	0.27	15 (20%)	26 (16.3%)	0.48	16 (21.1%)	25 (15.7%)	0.31	10 (21.7%)	31 (16.4%)	0.39
No	63 (78.8%)	131 (84.5%)		60 (80%)	134 (83.7%)		60 (78.9%)	134 (84.3%)		36 (78.3%)	158 (83.6%)	
Charlson Comorbidity Index (*n* = 216)	5.5 ± 1.7	5 ± 1.8	0.036	5.6 ± 1.9	4.9 ± 1.7	0.015	5.6 ± 1.8	4.9 ± 1.7	0.0073	5.8 ± 1.7	5.0 ± 1.8	0.011
Baseline Modified Borg Dyspnea Scale >2 (*n* = 237)												
Yes	19 (23.8%)	24 (15.3%)	0.11	15 (19.5%)	28 (17.5%)	0.71	16 (20.5%)	27 (17%)	0.51	11 (23.9%)	32 (16.7%)	0.26
No	61 (76.2%)	133 (84.7%)		62 (80.5%)	132 (82.5%)		62 (78.5%)	132 (83%)		35 (76.1%)	159 (83.3%)	
FEV1 (% of predicted) (*n* = 237)	77.4 ± 18.4	79.3 ± 16.8	0.45	75.1 ± 16.8	80.4 ± 17.4	0.026	75.2 ± 17.4	80.4 ± 17.1	0.029	74.1 ± 18.0	79.8 ± 17.0	0.051
FEV1/FVC ratio (*n* = 234)	69.7 ± 13.1	71.5 ± 13.0	0.31	71.7 ± 14.4	70.4 ± 12.3	0.52	71.7 ± 14.5	70.4 ± 12.3	0.51	68.2 ± 13.6	71.5 ± 12.8	0.12
Predictive postoperative FEV1 (ppoFEV1) (*n* = 182)	50.6 ± 9.3	50.3 ± 12.3	0.41	50.4 ± 10.9	50.4 ± 11.6	0.79	50.1 ± 10.7	50.5 ± 11.6	0.86	50.3 ± 9.5	50.4 ± 11.7	0.68
KCO (% of predicted) (*n* = 100)	78.8 ± 28.8	80.5 ± 17.8	0.091	79.0 ± 25.2	80.4 ± 20.5	0.28	79.3 ± 25.4	80.2 ± 20.3	0.33	77.3 ± 30.4	80.5 ± 19.5	0.059
Induction chemotherapy (*n* = 235)												
Yes	21 (26.3%)	50 (32.3%)	0.34	21 (28.0%)	50 (31.3%)	0.61	23 (30.3%)	48 (30.2%)	0.99	9 (19.6%)	62 (32.8%)	0.079
No	59 (73.7%)	105 (67.7%)		54 (72.0%)	110 (68.7%)		53 (69.7%)	111 (69.8%)		37 (80.4%)	127 (67.2%)	
Induction radiotherapy (*n* = 235)												
Yes	0	4 (2.6%)	0.36	0	4 (2.5%)	0.4	0	4 (2.5%)	0.39	0	4 (2.1%)	0.72
No	80 (100%)	141 (97.4%)		75 (100%)	156 (97.5%)		76 (100%)	155 (97.5%)		46 (100%)	185 (97.9%)	
Histology (*n* = 238):												
Adenocarcinoma	19 (23.5%)	57 (36.3%)	0.37	22 (28.2%)	54 (33.8%)	0.89	23 (29.1%)	53 (33.3%)	0.97	8 (17.0%)	68 (35.6%)	0.17
No-Adenocarcinoma	62 (76.5%)	100 (63.7%)		56 (71.8%)	106 (66.2%)		56 (70.9%)	106 (66.7%)		39 (8.5%)	123 (7.3%)	
Pathologic stage (*n* = 238)												
0	-	1 (0.6%)		-	1 (0.6%)	0.45	-	1 (0.6%)	0.36	-	1 (0.5%)	0.63
I	10 (12.3%)	9 (5.7%)	0.38	9 (11.5%)	10 (6.3%)		9 (11.4%)	10 (6.3%)		7 (14.9%)	12 (6.3%)	
II	19 (23.5%)	35 (22.3%)		16 (20.5%)	38 (23.8%)		20 (25.3%)	34 (21.4%)		10 (21.3%)	44 (23.0%)	
III	50 (61.7%)	105 (66.9%)		52 (66.7%)	103 (64.4%)		49 (62.0%)	106 (66.7%)		29 (61.7%)	126 (66.0%)	
IV	2 (2.5%)	7 (4.5%)		1 (1.3%)	8 (5.0%)		1 (1.3%)	8 (5.0%)		1 (2.1%)	8 (4.2%)	
pT (*n* = 237)												
T0	1 (1.2%)	1 (0.7%)	0.42	-	2 (1.3%)	0.45	-	2 (1.2%)	0.063	-	2 (1.0%)	0.041
T1	10 (12.3%)	13 (8.3%)		14 (18.2%)	9 (5.6%)		13 (16.7%)	10 (6.3%)		9 (19.1%)	14 (7.4%)	
T2	19 (23.5%)	44 (28.2%)		15 (19.5%)	48 (30.0%)		16 (20.5%)	47 (29.6%)		10 (21.3%)	53 (27.9%)	
T3	29 (35.8%)	68543.6%)		29 (37.6%)	68 (42.5%)		30 (38.5%)	67 (42.1%)		14 (29.8%)	83 (43.7%)	
T4	22 (27.2%)	30 (19.2%)		19 (24.7%)	33 (20.6%)		19 (24.3%)	33 (20.8%)		14 (29.8%)	38 (20.0%)	
pN (*n* = 238)												
N0	23 (28.4%)	31 (19.7%)	0.3	21 (26.9%)	33 (20.6%)	0.19	23 (29.1%)	31 (19.5%)	0.068	14 (29.8%)	40 (20.9%)	0.23
N1	31 (38.3%)	64 (40.8%)		37 (43.6%)	61 (38.1%)		34 (43.0%)	61 (38.4%)		20 (42.5%)	75 (39.3%)	
N2	27 (33.3%)	62 (39.5%)		23 (29.5%)	66 (41.3%)		22 (27.9%)	67 (42.1%)		13 (27.7%)	76 (39.8%)	
pM (*n* = 238)												
Mx	-	1 (0.6%)	0.72	-	1 (0.6%)	0.47	-	1 (0.6%)	0.45	-	1 (0.5%)	0.85
M0	79 (97.5%)	149 (95.0%)		77 (98.7%)	151 (94.4%)		78 (98.7%)	150 (94.4%)		46 (97.9%)	182 (95.3%)	
M1	2 (2.5%)	7 (4.4%)		1 (1.3%)	8 (5.0%)		1 (1.3%)	8 (45.0%)		1 (2.1%)	8 (4.2%)	

**Table 4:** Correlations of sarcopenia (according to the different muscles or muscular groups with fat exclusion) with different clinical and pathological parameters).

Features	Fat Excluded Psoas Sarcopenia		*p*-Value	Fat Excluded Parietal Muscle Sarcopenia		*p*-Value	Fat Excluded Total Muscle Area Sarcopenia		*p*-Value	Fat Excluded Sarcopenia of Both Psoas and Parietal Muscles		*p*-Value
	Yes	No		Yes	No		Yes	No		Yes	No	
Age	65.4 ± 9.9	61.4 ± 10.3	0.0084	65.4 ± 11.3	61.7 ± 9.6	0.0018	65.4 ± 11.2	61.7 ± 9.5	0.0019	66.3 ± 10.2	62.1 ± 10.2	0.0046
Men	56 (69.1%)	113 (72.0%)	0.65	56 (70.0%)	113 (72.0%)	0.75	56 (69.1%)	113 (72.0%)	0.65	36 (73.5%)	133 (70.4%)	0.67
Women	25 (30.9%)	44 (28.0%)		24 (30.0%)	44 (28.0%)		25 (30.9%)	44 (28.0%)		13 (26.5%)	56 (29.6%)	
Smokers (*n* = 291)												
Yes	73 (90.1%)	144 (92.3%)	0.57	67 (84.8%)	149 (94.9%)	0.0086	69 (86.3%)	148 (94.3%)	0.036	4 (83.7%)	176 (93.6%)	0.052
No	8 (9.9%)	12 (7.7%)		12 (15.2%)	8 (5.1%)		11 (13.7%)	9 (5.7%)		8 (16.3%)	12 (6.4%)	
Cumulative tobacco consumption	43.8 ± 20.9	43.54 ± 22.6	0.96	41.2 ± 24.4	44.8 ± 21.0	0.14	40.6 ± 23.7	45.1 ± 21.2	0.086	47.8 ± 24.78	42.6 ± 21.4	0.2
(Pack/Years) (*n* = 291)												
Right side	37 (45.7%)	71 (45.2%)	0.95	42 (52.5%)	66 (42.0%)	0.13	42 (51.9%)	66 (42.0%)	0.15	26 (53.1%)	82 (43.4%)	0.23
Left side	44 (54.3%)	86 (54.8%)		38 (47.5%)	91 (58.0%)		39 (48.1%)	91 (58.0%)		23 (46.9%)	107 (56.6%)	
Weight (kg)	62.4 ± 10.3	75.5 ± 13.5	<0.0000001	59.8 ± 9.4	75.9 ± 12.3	<0.0000001	59.7 ± 9.3	75.9 ± 12.4	<0.0000001	58.4 ± 8.8	73.5 ± 13.1	<0.0000001
Height (cm)	168.2 ± 8.8	169.4 ± 9.4	0.48	166.6 ± 10.0	170.2 ± 8.5	0.027	166.5 ± 9.9	170.3 ± 8.5	0.015	168.1 ± 9.3	169.2 ± 9.2	0.63
BMI (kg/m^2^)	22.3 ± 3.5	25.9 ± 4.0	<0.0000001	21.6 ± 3.3	26.2 ± 3.7	<0.0000001	21.7 ± 3.3	26.2 ± 3.8	<0.0000001	20.8 ± 3.1	25.4 ± 4.0	<0.0000001
Obesity												
Yes	2 (2.5%)	28 (18.2%)	0.00067	0	30 (19.5%)	0.000026	0	30 (19.5%)	0.000024	0	30 (16.1%)	0.0029
No	78 (97.5%)	126 (81.8%)		79 (100%)	124 (80.5%)		80 (100%)	124 (80.5%)		48 (100)	156 (83.9%)	
Diabetes (*n* = 235)												
Yes	8 (9.9%)	12 (7.8%)	0.59	7 (9.0%)	13 (8.3%)	0.87	8 (10.1%)	12 (7.7%)	0.53	5 (10.2%)	15 (8.1%)	0.85
No	73 (90.1%)	142 (92.2%)		71 (91.0%)	143 (91.7%)		71 (89.9%)	146 (92.83%)		44 (89.8%)	171 (91.9%)	
Hypertension (*n* = 235)												
Yes	30 (37.0%)	53 (34.4%)	0.69	29 (37.2%)	54 (34.6%)	0.7	30 (38.0%)	53 (34.0%)	0.54	17 (34.7%)	66 (35.5%)	0.92
No	51 (63.0%)	101 (65.6%)		49 (62.8%)	102 (65.4%)		49 (62.0%)	103 (66.0%)		32 (65.3%)	120 (65.5%)	
Ischemic heart disease (*n* = 235)												
Yes	16 (19.7%)	25 (16.2%)	0.5	16 (20.5%)	25 (16.0%)	0.39	17 (21.5%)	24 (15.4%)	0.24	10 (20.4%)	31 (16.7%)	0.54
No	65 (80.3%)	129 (83.8%)		62 (79.5%)	131 (84.0%)		62 (78.5%)	132 (84.6%)		39 (79.6%)	155 (83.3%)	
Charlson Comorbidity Index (*n* = 216)	5.4 ± 1.7	5 ± 1.8	0.066	5.4 ± 1.9	5.0 ± 1.7	0.16	5.4 ± 1.8	5.0 ± 1.8	0.14	5.6 ± 1.7	5.0 ± 1.8	**0.038**
Baseline Modified Borg Dyspnea Scale >2 (*n* = 237)												
Yes	20 (25.0%)	23 (14.7%)	0.051	16 (20.3%)	27 (17.2%)	0.57	16 (20.0%)	27 (17.2%)	0.6	13 (27.1%)	30 (15.9%)	0.072
No	60 (75.0%)	134 (85.3%)		63 (79.7%)	130 (82.8%)		64 (80.0%)	130 (82.8%)		35 (72.9%)	159 (84.1%)	
FEV1 (% of predicted) (*n* = 237)	76.7 ± 17.7	79.7 ± 17.1	0.22	76.5 ± 17.0	79.9 ± 17.5	0.16	76.9 ± 17.6	79.6 ± 17.2	0.27	74.0 ± 17.3	79.9 ± 17.2	**0.033**
FEV1/FVC ratio (*n* = 234)	69.5 ± 12.4	71.6 ± 13.3	0.37	71.4 ± 13.0	70.6 ± 13.1	0.64	71.6 ± 13.3	70.4 ± 12.9	0.5	67.8 ± 11.6	71.7 ± 13.3	0.14
Predictive postoperative FEV1 (ppoFEV1) (*n* = 182)	49.6 ± 9.5	50.8 ± 12.2	0.9	50.9 ± 10.8	50.2 ± 11.7	0.5	50.5 ± 10.4	50.3 ± 11.8	0.65	49.0 ± 10.6	50.7 ± 11.5	0.39
KCO (% of predicted) (*n* = 100)	77.8 ± 28.7	81.2 ± 17.2	**0.046**	77.0 ± 25.0	81.7 ± 20.4	0.055	76.3 ± 25.7	82.3 ± 19.3	**0.028**	75.7 ± 28.9	81.4 ± 19.2	**0.022**
Induction chemotherapy (*n* = 235)												
Yes	22 (27.2%)	49 (31.8%)	0.46	26 (33.3%)	45 (28.9%)	0.48	26 (32.9%)	45 (28.9%)	0.52	12 (24.5%)	59 (31.7%)	0.33
No	59 (72.8%)	105 (68.2%)		52 (66.7%)	111 (71.1%)		53 (67.1%)	111 (71.1%)		37 (75.5%)	127 (68.3%)	
Induction radiotherapy (*n* = 235)												
Yes	0	4 (2.6%)	0.35	0	4 (2.6%)	0.37	0	4 (2.6%)	0.37	0	4 (2.1%)	0.68
No	81 (100%)	150 (97.4%)		78 (100%)	152 (97.4%)		79 (100%)	152 (97.4%)		49 (100%)	182 (97.9%)	
Histology (*n* = 238):												
Adenocarcinoma	22 (27.2%)	54 (34.4%)	0.75	27 (33.8%)	48 (30.6%)	0.92	26 (32.1%)	50 (31.8%)	0.91	13 (26.5%)	63 (33.3%)	0.79
No-Adenocarcinoma	59 (72.8%)	103 (65.6%)		53 (66.2%)	109 (69.4%)		55 (67.9%)	107 (68.2%)		36 (73.5%)	126 (66.7%)	
Pathologic stage (*n* = 238)												
0	-	1 (0.6%)	0.38	-	1 (0.6%)	0.41	-	1 (0.6%)	0.41	-	1 (0.5%)	0.66
I	10 (12.3%)	9 (5.7%)		9 (11.2%)	10 (6.4%)		9 (11.1%)	10 (6.4%)		7 (14.3%)	12 (6.4%)	
II	19 (23.5%)	35 (22.3%)		20 (25.0%)	34 (21.7%)		20 (24.7%)	34 (21.7%)		10 (20.4%)	44 (23.3%)	
III	50 (61.7%)	105 (66.9%)		50 (62.5%)	104 (66.2%)		51 (63.0%)	104 (66.2%)		31 (63.3%)	124 (65.6%)	
IV	2 (2.5%)	7 (4.5%)		1 (1.3%)	8 (5.1%)		1 (1.2%)	8 (5.1%)		1 (2.0%)	8 (4.2%)	
pT (*n* = 237)												
T0	1 (1.2%)	1 (0.7%)	0.42	-	2 (1.3%)	0.4	-	2 (1.3%)	0.14	-	2 (1.0%)	0.2
T1	10 (12.3%)	13 (8.3%)		11 (13.9%)	12 (7.6%)		12 (15.0%)	11 (7.0%)		8 (16.3%)	15 (8.0%)	
T2	19 (23.5%)	44 (28.2%)		19 (24.1%)	44 (28.0%)		18 (22.5%)	45 (28.7%)		11 (22.4%)	52 (27.7%)	
T3	29 (35.8%)	68 (43.6%)		30 (37.9%)	67 (42.7%)		29 (36.2%)	68 (43.3%)		16 (32.7%)	81 (43.1%)	
T4	22 (27.2%)	30 (19.2%)		19 (24.1%)	32 (20.4%)		21 (26.3%)	31 (19.7%)		14 (28.6%)	38 (20.2%)	
pN (*n* = 238)												
N0	24 (29.6%)	30 (19.1%)	0.18	24 (30.0%)	30 (19.1%)	**0.05**	22 (27.2%)	32 (20.4%)	0.27	16 (32.7%)	38 (20.1%)	0.13
N1	29 (35.8%)	66 (42.0%)		34 (42.5%)	61 (38.9%)		34 (42.0%)	61 (38.9%)		19 (38.8%)	76 (40.2%)	
N2	28 (34.6%)	61 (38.9%)		22 (27.5%)	66 (42.0%)		25 (30.8%)	64 (40.8%)		14 (28.6%)	75 (39.7%)	
pM (*n* = 238)												
Mx	-	1 (0.6%)	0.72	-	1 (0.6%)	0.43	-	1 (0.6%)	0.43	-	1 (0.5%)	0.83
M0	79 (97.5%)	149 (95.0%)		79 (98.7%)	148 (94.3%)		80 (98.7%)	148 (94.3%)		48 (97.9%)	180 (95.3%)	
M1	2 (2.5%)	7 (4.4%)		1 (1.3%)	8 (5.1%)		1 (1.3%)	8 (5.1%)		1 (2.1%)	8 (4.2%)	

**Table 5:** Five-year survival according to clinical and pathologic factors: univariate analysis.

Features	5 Years Overall Survival (95% CI) Total Sample = 238	*p*-Value
Age (median 64 years)		0.0019
≤64	49.6 (41.05–58.19)	
>64	27.0 (19.64–35.95)	
Sex:		0.0011
Women	56.5 (44.79–67.57)	
Men	32.0 (25.39–39.32)	
Current/Never Smokers (*n* = 227)		0.21
Current	37.3 (31.16–43.93)	
Never	55 (34.21–74.18)	
Cumulative tobacco consumption (Pack/Years) (*n* = 220) (median 40)		0.96
≤40	37.5 (29–35)	
>40	39 (30.02–48.8)	
Right/Left side		0.22
Right	35.2 (26.83–44.56)	
Left	42.3 (34.16–50.90)	
Underweight	22.2 (6.32–54.74)	0.0096
Normal weight/Overweight/Obesity	39.6 (33.39–46.07)	
Diabetes Yes/No (*n* = 235)		0.58
Yes	30 (14.55–51.9)	
No	35.5 (33.24–46.2)	
Hypertension Yes/No (*n* = 235)		0.017
Yes	27.7 (19.23–38.16)	
No	44.7 (3.06–52.67)	
Ischemic heart disease Yes/No (*n* = 235)		0.000076
Yes	14.6 (6.88–28.4)	
No	43.8 (37.02–50.85)	
Charlson Comorbidity Index (*n* = 216)		0.005
≤5	46.5 (38.01–55.11)	
>5	25.8 (17.88–35.8)	
Baseline Modified Borg Dyspnea Scale >2 (*n* = 237) Yes/No		0.18
Yes	48.8 (34.62–63.25)	
No	37.1 (30.63–44.1)	
FEV1 (% of predicted) (*n* = 237)		0.34
≥80	42.7 (33.28–52.7)	
<80	36.2 (28.7–44.37)	
FEV1/FVC ratio (*n* = 234)		0.19
≥70	41.9 (33.7–50.49)	
<70	36.2 (27.64–45.72)	
Predictive postoperative FEV1 (ppoFEV1) (*n* = 182)		0.074
≤50	34.3 (25.82–43.95)	
>50	46.3 (35.75–57.1)	
KCO (% of predicted) (*n* = 100)		0.32
≤80	32.7 (21.81–45.90)	
>80	40.9 (27.69–55.59)	
Induction chemotherapy Yes/No (*n* = 235)		0.068
Yes	29.6 (20.23–41.02)	
No	42.7 (35.37–50.34)	
Induction radiotherapy Yes/No (*n* = 235)		0.068
Yes	29.6 (20.23–41.02)	
No	42.7 (35.37–50.34)	
Histology (*n* = 238):		0.9
Adenocarcinoma	39.5 (29.25–50.71)	
No-adenocarcinoma	38.9 (31.72–46.57)	
Pathologic stage (*n* = 238)		0.015
I–II	45.2 (34.31–56.58)	
IIIA	20.6 (31.71–50.08)	
IIIB	30.6 (19.52–44.53)	
IV	11.1 (1.99–43.5)	
pT (*n* = 237)		
T1-T2	44.2 (34.16–54.71)	0.068
T3-T4	34.9 (27.71–48.85)	
pN (*n* = 238)		0.2
N0	42.6 (30.33–55.84)	
N1	44.2 (34.64–54.23)	
N2	31.5 (22.75–41.7)	
pM (*n* = 238)		0.003
M0	40.4 (34.19–50.71)	
M1	12.5 (2.24–47.09)	

**Table 6:** Five-year survival according to morphometric measurements: univariate analysis.

Features	5 Years Overall Survival (95% CI) Total Sample = 238	*p*-Value
Total psoas area with fat exclusion (FE-TPA)		0.15
Yes	33.3 (24.03–44.15)	
No	42.0 (34.6–49.86)	
Total psoas area (TAP)		0.034
Yes	30.9 (21.86–41.6)	
No	43.3 (35.81–51.13)	
Total parietal muscle area with fat exclusion (FE-TPMA)		0.054
Yes	33.3 (23.87–44.36)	
No	41.9 (34.51–49.62)	
Total parietal muscle area (TPMA)		0.005
Yes	27.5 (18.92–38.14)	
No	44.6 (37.03–52.4)	
Total muscle area with fat exclusion (FE-TMA)		0.017
Yes	30.4 (21.34–41.23)	
No	43.4 (35.94–51.17)	
Total muscle area (TMA)		0.018
yes	29.6 (20.79–40.31)	
No	43.9 (36.42–51.77)	
Sarcopenia on both (FE-SARC) with fat exclusion		0.041
Yes	29.8 (18.65–43.58)	
No	41.4 (34.61–48.45)	
Sarcopenia on both (SARC)		0.00024
Yes	20.4 (11.48–33.64)	
No	43.9 (37.03–51.04)	

**Table 7:** Multivariate analysis. Two stepwise Cox model assessing the independent prognostic value of factors identified at univariate analysis are displayed. In Model 1, total sarcopenia on both psoas and parietal muscles was introduced. In Model 2, TMA was entered. In both models, sex, age (<64 vs. > 64 years), Charlson Comorbidity Index (CCI) (<5 vs. >5), BMI (underweight vs. others), and pathologic stage (four classes: I–II, IIIA, IIIB, and IV) were also introduced.

Model 1		Relative Risk	95% IC	*p*-Value
Pathologic stage	I–II			0.0013
	IIIA	1.39	(1.14–1.70)	
	IIIB	1.93	(1.29–2.89)	
	IV	2.59	(1.47–4.92)	
Sex	Men	1.95	(1.30–2.92)	0.0012
Sarcopenia	Total	1.71	(1.17–2.52)	0.006
Age	>64 years	1.48	(1.06–2.06)	0.04
**Model 2**				
Pathologic stage	I–II			0.0011
	IIIA	1.39	(1.14–1.70)	
	IIIB	1.94	(1.30–2.89)	
	IV	2.70	(1.49–4.91)	
Sex	Men	1.89	(1.26–2.85)	0.0022
Sarcopenia	Total	1.54	(1.10–2.16)	0.0012
Age	>64 years	1.48	(1.06–2.06)	0.04
CCI	>5	1.51	(1.08–2.10)	0.0015

## Data Availability

Available upon reasonable request. Data sharing not applicable.

## Abbreviations

ADK	adenocarcinoma
ARDS	acute respiratory distress syndrome
ARF	acute respiratory failure
CCI	Charlson Comorbidity Index
COPD	chronic obstructive pulmonary disease
CT	computed tomography
FEV1	forced expiratory volume
FVC	forced vital capacity
MV	mechanic ventilation
PPO FEV1	predictive postoperative FEV1
TMA	total muscle area
TPA	total psoas area
TPMA	total parietal muscle area
SCC	squamous cell carcinoma
SD	standard deviation
SMI	skeletal muscle index

## References

[1] Sugarbaker D.J., Haywood-Watson R.J., Wald O. (2016). Pneumonectomy for Non-Small Cell Lung Cancer. Surg. Oncol. Clin. N. Am..

[2] Alifano M., Cusumano G., Strano S., Magdeleinat P., Bobbio A., Giraud F., Lebeau B., Regnard J.-F. (2009). Lobectomy with pulmonary artery resection: Morbidity, mortality, and long-term survival. J. Thorac. Cardiovasc. Surg..

[3] Janet-Vendroux A., Loi M. (2015). Which is the Role of Pneumonectomy in the Era of Parenchymal-Sparing Procedures? Early/Long-Term Survival and Functional Results of a Single-Center Experience. Lung.

[4] Blanc K., Dechartres A., Zaimi R., Lefebvre A., Janet-Vendroux A., Fournel L., Dermine H., Lorut C., Becanne X., Hamelin-Canny E. (2018). Patients experiencing early acute respiratory failure have high postoperative mortality after pneumonectomy. J. Thorac. Cardiovasc. Surg..

[5] Blanc K., Zaimi R., Dechartres A., Lefebvre A., Janet-Vendroux A., Hamelin-Canny E., Roche N., Alifano M., Rabbat A. (2018). Early acute respiratory distress syndrome after pneumonectomy: Presentation, management, and short- and long-term outcomes. J. Thorac. Cardiovasc. Surg..

[6] Daffrè E., Prieto M. (2020). Normalized pulmonary artery diameter predicts occurrence of postpneumonectomyrespiratory failure, ARDS and mortality. Cancers.

[7] Detterbeck F.C., Postmus P.E., Tanoue L.T. (2013). The Stage Classification of Lung Cancer: Diagnosis and Management of Lung Cancer, American College of Chest Physicians evidence-based clinical practice guidelines. Chest.

[8] Hervochon R., Bobbio A. (2017). Body Mass Index and Total Psoas Area Affect Outcomes in Patients Undergoing Pneumonectomy for Cancer. Ann. Thorac. Surg..

[9] Icard P., Iannelli A. (2018). Sarcopenia in resected non-small cell lung cancer: Let’s move to patient-directed strategies. J. Thorac. Dis..

[10] Cruz-Jentoft A.J., Bahat G. (2019). Sarcopenia: Revised European consensus on definition and diagnosis. Age Ageing..

[11] Martini K., Chassagnon G., Fournel L., Prieto M., Hoang-Thi T.-N., Halm N., Bobbio A., Revel M.-P., Alifano M. (2020). Sarcopenia as independent risk factor of postpneumonectomy respiratory failure, ARDS and mortality. Lung Cancer.

[12] Foldyna B., Troschel F.M., Addison D., Fintelmann F.J., Elmariah S., Furman D., Eslami P., Ghoshhajra B., Lu M.T., Murthy V.L. (2018). Computed tomography-based fat and muscle characteristics are associated with mortality after transcatheter aortic valve replacement. J. Cardiovasc. Comput. Tomogr..

[13] Icard P., Schussler O., Loi M., Bobbio A., Lupo A.M., Wislez M., Iannelli A., Fournel L., Damotte D., Alifano M. (2020). Pre-Disease and Pre-Surgery BMI, Weight Loss and Sarcopenia Impact Survival of Resected Lung Cancer Independently of Tumor Stage. Cancers.

[14] Goldstraw P., Chansky K., Crowley J., Rami-Porta R., Asamura H., Eberhardt W.E., Nicholson A.G., Groome P., Mitchell A., Bolejack V. (2016). The IASLC Lung Cancer Staging Project: Proposals for Revision of the TNM Stage Groupings in the Forthcoming (Eighth) Edition of the TNM Classification for Lung Cancer. J. Thorac. Oncol..

[15] https://www.insee.fr.

[16] Hasselager R., Gögenur I., Hasselager R. (2014). Core muscle size assessed by perioperative abdominal CT scan is related to mortality, postoperative complications, and hospitalization after major abdominal surgery: A systematic review. Langenbecks Arch. Surg..

[17] Suzuki Y., Okamoto T., Fujishita T., Katsura M., Akamine T., Takamori S., Morodomi Y., Tagawa T., Shoji F., Maehara Y. (2016). Clinical implications of sarcopenia in patients undergoing complete resection for early non-small cell lung cancer. Lung Cancer.

[18] Madariaga M.L.L., Troschel F.M., Best T.D., Knoll S.J., Gaissert H.A., Fintelmann F.J. (2020). Low Thoracic Skeletal Muscle Area Predicts Morbidity After Pneumonectomy for Lung Cancer. Ann. Thorac. Surg..

[19] Alifano M., Mansuet-Lupo A. (2014). Systemic inflammation, nutritional status and tumor immune microenvironment determine outcome of resected non-small cell lung cancer. PLoS ONE.

[20] Jagoe R.T., Redfern C.P.F., Roberts R.G., Gibson G.J., Goodship T.H.J. (2002). Skeletal muscle mRNA levels for cathepsin B, but not components of the ubiquitin‒proteasome pathway, are increased in patients with lung cancer referred for thoracotomy. Clin. Sci..

[21] Argilés J.M., Busquets S., Stemmler B., López-Soriano F.J. (2014). Cancer cachexia: Understanding the molecular basis. Nat. Rev. Cancer.

[22] Taguchi S., Akamatsu N., Nakagawa T., Gonoi W., Kanatani A., Miyazaki H., Fujimura T., Fukuhara H., Kume H., Homma Y. (2016). Sarcopenia Evaluated Using the Skeletal Muscle Index Is a Significant Prognostic Factor for Metastatic Urothelial Carcinoma. Clin. Genitourin. Cancer.

